# Beyond maternal health: a gendered analysis of the associations between publicly-funded health insurance, service utilisation and financial protection among Indian women

**DOI:** 10.1186/s12913-026-15704-7

**Published:** 2026-10-05

**Authors:** Susanne Ziegler, Swati Srivastava, Divya Parmar, Sharmishtha Basu, Nishant Jain, Manuela De Allegri

**Affiliations:** 1 Heidelberg Institute of Global Health, Medical Faculty and University Hospital, Heidelberg University, Im Neuenheimer Feld 130.3, 69120 Heidelberg, Germany; 2 Deutsche Gesellschaft für Internationale Zusammenarbeit (GIZ) GmbH, Friedrich-Ebert-Allee 32+36, 53113 Bonn, Germany; 3 Public Health Foundation of India, New Delhi, 110030 India; 4 Department of Population Health Sciences, School of Life Course and Population Sciences, Weston Education Centre, King’s College London, Cutcombe Road, London, SE5 9RJ UK; 5 Deutsche Gesellschaft für Internationale Zusammenarbeit (GIZ) GmbH, B5/1 Safdarjung Enclave, New Delhi, 110029 India

**Keywords:** Universal health coverage, Health insurance, Maternal health, Financial protection, India, RSBY, PM-JAY

## Abstract

**Background:**

There is a lack of evidence regarding the role of universal health coverage (UHC) schemes, such as publicly-funded health insurance (PFHI), on women’s utilisation of health services and financial protection, particularly for conditions beyond maternal care. To address this gap, we conducted a gender analysis of healthcare utilisation and out-of-pocket expenditure (OOPE) among poor and vulnerable women in India, focusing on India’s first large national PFHI scheme, *Rashtriya Swasthya Bima Yojana* (RSBY).

**Methods:**

We analysed data from a cross-sectional household survey in eight Indian states to compare the utilisation and OOPE of women and men among RSBY-eligible families. We used bivariate analysis, multivariate logistic and GLM regressions, controlled for demographic and socio-economic characteristics and examined maternal health and non-maternal health services separately. Statistical significance was assessed at 10%, 5% and 1% levels.

**Results:**

Independent of enrolment in RSBY, women had a higher use of hospitalisation services than men, but only when services for maternal health were included in the analysis (women: 4%, men: 3.23%, *p* < 0.001). Enrolment in RSBY did not influence the decision to be hospitalised for either men or women. However, suggestive evidence indicates that it was associated with reduced OOPE for women who were hospitalised for services other than maternal health (coef = -0.25; *p* = 0.087).

**Conclusion:**

The findings highlight the potential benefits of PFHIs in providing financial protection for women beyond maternal health. To further improve access to healthcare for poor and vulnerable women, it is imperative that their healthcare needs are considered throughout their lifecycle when health policy interventions are designed and implemented. We recommend further research on the gendered effects of PFHIs, as defined in this paper, and on how different bases for entitlement influence equitable access to health care and financial protection. Findings should feed into the reform of tax-funded, non-contributory financing arrangements with purchaser-provider splits and explicit benefit packages in India and other LMICs, ensuring that these financing arrangements are gender-responsive, universally designed and implemented and ultimately reach all poor and vulnerable individuals.

**Supplementary Information:**

The online version contains supplementary material available at https://doi.org/10.1186/s12913-026-15704-7.

## Introduction

Universal health coverage (UHC) entails that all people should have access to the essential health services they need without suffering from financial hardship [1]. Many people in low- and middle-income countries (LMICs) face difficulties in accessing such services due to persisting inequities in wealth, ethnicity, education, geographic location and sociocultural factors [2–4]. This is especially true for women, as gender norms and power relations that persist in many societies influence women’s ability to access healthcare services and ultimately their health outcomes [5]. To improve access to health services and increase financial protection in low-income settings, many LMICs have introduced publicly-funded health insurance (PFHI) schemes. Albeit different in design and implementation across countries, they all share the same goal, namely to improve access to healthcare for poor and vulnerable populations who are often excluded from other forms of insurance and social health protection coverage [6, 7]. An example for such a scheme is Thailand’s tax-funded Universal Coverage Scheme that has led to substantial reductions in out-of-pocket expenditure (OOPE), catastrophic health expenditure and medical impoverishment [8]. Similarly, Indonesia’s Jaminan Kesehatan Nasional (JKN), financed through contributions and government subsidies for low-income populations, has been associated with increased utilisation of healthcare services [9].

PFHI have been recommended as one of the most equitable routes to achieving UHC [10, 11]. Systematic reviews highlight that different types of health insurance, including PFHIs, have a positive effect on improving access to health services and reducing OOPE for healthcare [12–14]. Nonetheless, specific evidence on the benefits accrued by women, both in terms of increased health service utilisation and financial protection, remains extremely limited. This lack of evidence may be derived from the assumption that a well-functioning health system progressing toward UHC will inherently be equitable and gender-neutral [15, 16]. The limited evidence available regarding the potential effects of PFHIs on women focuses mainly on their utilisation of maternal and child health (MCH) services and confirms that different types of health insurance improve women’s access to these services [17–20]. This focus is not surprising, as health policy reforms, healthcare delivery systems and research have long prioritised women’s reproductive capacities with significant improvements in MCH outcomes: for instance, the global maternal mortality ratio dropped by 38% between 2000 and 2017 [21].

Nonetheless, women’s health needs are much broader than just MCH and reproductive health, and this should be acknowledged throughout women’s entire lifecycle. This is especially important because, for example, non-communicable diseases (NCDs) have become the leading cause of death among women, accounting for 73% of all deaths of women worldwide [3]. In addition, women living in more gender-inequitable societies are more likely to suffer from the double burden of infectious diseases (IDs) and NCDs, as their dependency on men and other household members often hinders them from seeking treatment [22].

The available evidence examining the benefits accrued by women enrolled in different types of health insurance beyond MCH services is limited to a handful of studies. Using data from the 2002-04 World Health Survey in 48 LMICs, El-Sayed et al. (2015) revealed that health insurance improved the likelihood of women receiving treatment for NCDs, but the study did not differentiate between different types of insurance, such as publicly-funded, private or community-based health insurance [23]. Another study by Samarakoon and Parinduri (2015) found that enrolment in a PFHI in Indonesia did not increase women’s use of preventive and curative care [20]. To the best of our knowledge, no study to date has examined a reduction in OOPE specifically for women enrolled in PFHI accessing services beyond MCH. This implies that we do not have a full understanding of the potential for financial protection that PFHI can have for this population group.

The gaps observed at the global level are also reflected in the Indian context. India has a longstanding tradition of not only implementing PFHIs at the central and state levels, but also of initiating health programmes specifically targeted at women with a focus on MCH services, which resulted, among others, in the reduction of the maternal mortality ratio by 59% between 2000 and 2017 [21]. There is less focus on other services, although, for example, the prevalence of NCDs and IDs is higher for Indian women (63 per thousand persons and 8.32, respectively) than for men (47 and 8.2, respectively) [24, 25].

PFHIs in India provide a broad range of services, including for MCH, NCD and ID, and mainly cover the costs of hospitalisations. The *Rashtriya Swasthya Bima Yojana* (RSBY), operational from 2008 to 2018, is a PFHI which was introduced in order to improve access to healthcare services and reduce OOPE for poor and informally employed population groups. Available research on RSBY provides mixed results regarding women: overall enrolment in RSBY was largely gender-neutral, and households headed by women were more likely to enrol [26–28]. Numerous studies conclude that the scheme has had limited or no success in providing financial protection to beneficiaries and has even increased OOPE, but the results do not differentiate between men and women [29–34]. Research also suggests that access to health services improved for women under RSBY: once enrolled, women used services more often than men [29, 35], yet studies found that women used RSBY mainly for MCH services like deliveries [36, 37]. This led to reduced infant and under-five mortality, especially among urban poor households [37]. Other studies have specifically analysed the utilisation of MCH services under RSBY and revealed that RSBY reduced OOPE related to MCH services, but also revealed that effects could have been higher if beneficiaries had been better informed about the entitlements and benefits of the scheme [36, 38, 39]. To our knowledge, literature on the effect that PFHI can have on women when they utilise non-MCH services is lacking for India, including for RSBY.

Our brief review of the literature points to the need to build a more robust body of evidence on the benefits accrued by women given their enrolment in a PFHI, specifically measured in relation to an increased utilisation of healthcare services and increased financial protection. This is essential to inform more gender-sensitive health policies and remove financial barriers that women face when accessing services, especially in light of India’s shift in disease burden and the rising prevalence of NCDs among women [40].

Our work is thus set against this background and aims to fill this gap in knowledge by performing a gendered analysis of healthcare utilisation and related OOPE under RSBY across eight Indian states. Our objective is to examine the extent to which RSBY was linked to the same benefits for men and women, measured in terms of increased health service utilisation and reduced OOPE. In line with our intention to move beyond existing analyses of PFHI, which focus mostly on MCH matters, we examined the association of the scheme with service use, both including and excluding the utilisation of maternal health services. RSBY was subsumed under the larger *Pradhan Mantri Jan Arogya Yojana* (PM-JAY) in 2018, but it still presents an interesting historical research opportunity as there are many similarities in the objectives and implementation modalities of RSBY, PM-JAY, as well as other PFHIs active in other states [41]. Key characteristics of RSBY and PM-JAY, including both schemes’ design features and eligibility criteria, are available in the supplementary files, Table S1.

We trust that useful lessons for policy makers and implementers can be learned, given RSBY’s originality and the wide geographical scope of our sample. Our study is a sex-disaggregated analysis with gender-sensitive interpretation, using *sex* for distinctions that arise from the biological differentiation of being male or female, and *gender* to socially constructed roles, operationalised here as men and women. In this paper, we use the term PFHI to refer to tax-funded, non-contributory government health financing programmes that provide eligible populations with an explicit package of cashless health services through strategic purchasing of care from contracted healthcare providers. We use the term as a shorthand for this broader set of financing and purchasing arrangements, rather than to imply that the insurance label itself is the mechanism through which health and financial protection outcomes are achieved.

## Methods

### Study setting

RSBY and PM-JAY share several design features, including the provision of cashless inpatient care through empanelled public and private hospitals across India, while excluding outpatient services from coverage. Like PM-JAY, RSBY covered a broad range of inpatient procedures, diagnostics, and pre- and post-hospitalisation expenses, although neither scheme included outpatient or primary care services. However, PM-JAY substantially expanded the level of financial protection offered under RSBY: whereas RSBY provided annual coverage of up to INR 30,000 per household, PM-JAY increased this limit to INR 500,000 per family per year for secondary and tertiary inpatient care [35, 42].

The two schemes also differ in their eligibility and enrolment arrangements. RSBY targeted households living below the poverty line, including beneficiaries of programmes such as the Mahatma Gandhi National Rural Employment Guarantee Act and the Antyodaya Anna Yojana [28, 43]. Enrolment under RSBY required eligible households to pay an annual registration fee of INR 30, visit an enrolment station, and provide biometric information, after which they received a chip-based smart card to access services [28, 35, 44]. In contrast, PM-JAY identifies eligible households through deprivation and occupational criteria listed in the Socio-Economic Caste Census and does not require annual enrolment or registration fees [42].

A further distinction concerns family coverage. RSBY restricted enrolment to a maximum of five household members, including the head of household, spouse, and up to three dependents [28, 35, 45]. This cap, likely an enrolment deterrent, may have contributed to lower enrolment rates among women and female-headed households [46, 47], whereas other studies have reported higher enrolment rates in female-headed households [27, 28, 48]. PM-JAY removed this restriction entirely, imposing no cap on family size, thereby broadening access for larger households [42].

### Data sources and sampling

**Fig. 1:**
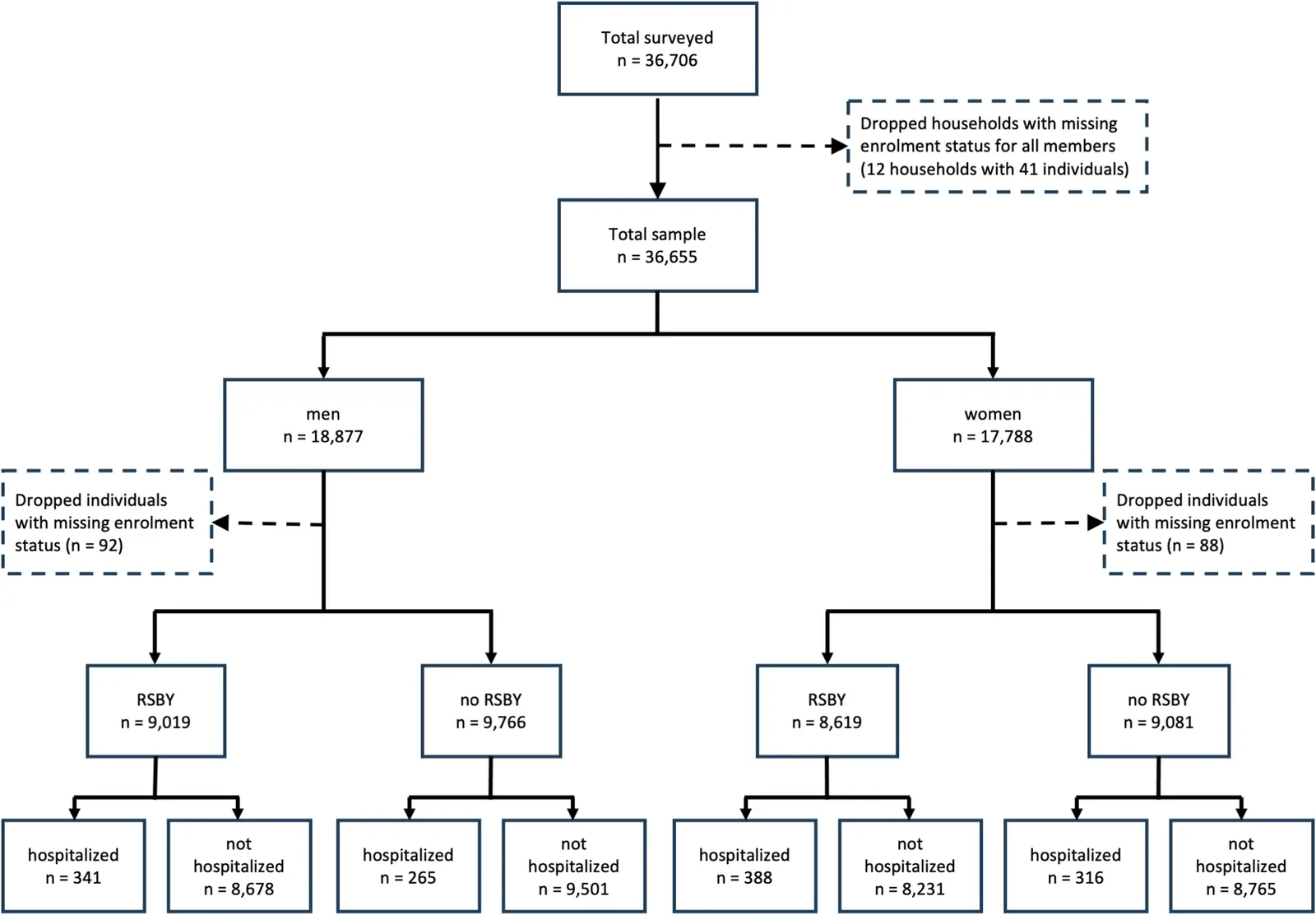
Composition of the study sample. Source: own depiction

This study draws on a 2014 cross-sectional household survey previously described by Ziegler et al. [28], conducted as part of a broader RSBY evaluation. The survey covered RSBY-eligible households in eight Indian states: Bihar, Gujarat, Kerala, Mizoram, Tripura, Uttarakhand, Uttar Pradesh and West Bengal. These states were purposively selected in consultation with the Indian central and state governments with the intention to capture variation in socio-demographic and economic contexts, as well as differences in the progress of RSBY implementation. For the present analysis, we used the anonymised individual- and household-level data from this survey. The sample was generated through a combination of multi-stage purposive and random sampling methods; the full sampling procedure is reported in Ziegler et al. [28]. Approximately 1,000 households were surveyed in each of Gujarat, Kerala, Mizoram and Tripura, and approximately 900 households in each of Bihar, Uttarakhand, Uttar Pradesh and West Bengal. The final sample comprised 7,609 households and 36,665 individuals. Figure 1 depicts the pathway to identify the composition of the final sample.

### Variables and their measurement

Table 1 provides an overview of the variables used in the analyses with their unit of measurement and distribution in the sample. The analysis took into consideration two outcome variables: (1) a binary variable *hospitalisation* denoting whether an individual was hospitalised (*yes*) or not (*no*) in the last 365 days; (2) *OOPE*, a continuous variable that denoted the OOPE (in INR) associated with the respective hospitalisation in the last 365 days. OOPE included direct medical expenditures, namely consultation fees, diagnostic tests, and medicines, and non-medical expenditures such as food and transportation costs. We found a strong right-tailed distribution and extreme outliers. As this is problematic for the regression models, we symmetrically winsorised the OOPE variable by replacing the lower and upper 3% of outliers with the respective lowest and highest values of the subsample (at the 3rd and 97th percentiles, respectively) [49]. This approach allowed us to keep all observations and prevent outliers from distorting the findings of the regression models. It has previously been applied for analyses with similar challenges [50, 51]. Sensitivity analysis using the unwinsorised data showed no changes in the significance of the key exposure variables, therefore supporting our approach (see supplementary files, Table S3). The key exposure variables were *sex* (male or female) and *enrolment in RSBY* (yes or no). To account for potential confounders, we included a set of covariates that captured the socio-economic, demographic and geographic characteristics of individuals and households.

**Table 1:** Used variables, their measurement and their distribution in the sample (in total and disaggregated by sex)

Variables	Measurement	Distribution					
		Total		Men		Women	
		n	%	n	%	n	%
**Outcome variables**							
individuals hospitalised	0 = no	35,344	96.40	18,267	51.68	17,077	48.32
	1 = yes	1321	3.60	610	46.18	711	53.82
		Mean	SD	Mean	SD	Mean	SD
OOPE	Indian Rupees (continuous)	3816.53	5057.91	4236.27	5390.45	3452.60	4724.80
**Key exposure variables**							
sex	1 = male	18,877	51.49	-		-	
	2 = female	17,788	48.51	-		-	
enrolment in RSBY	1 = yes	17,638	48.34	9019	51.13	8619	48.87
	2 = no	18,847	51.66	9766	51.82	9081	48.18
	*missing*	*180*					
**Covariates individual level**							
age	1 = 0–14	10,312	28.12		51.49		48.51
	2 = 15–29	10,615	28.95		52.83		47.17
	3 = 30–49	9381	25.59		49.73		50.27
	3 = 50+	6357	17.34		51.82		48.18
relationship to hoh	1 = head of household	7609	20.75		88.92		11.08
	2 = spouse	6281	17.13		1.80		98.20
	3 = child	16,709	45.57		58.31		41.69
	4 = others	6066	16.54		37.17		62.83
education	1 = no education	12,154	33.15		43.36		56.64
	2 = up to 7 years	12,921	35.24		53.54		46.46
	3 = 8 years and above	11,590	31.61		57.71		42.29
occupation	1 = farming	4794	13.08		81.50		18.50
	2 = labourer/ daily wages	5436	14.83		84.44		15.56
	3 = other occupation	2266	6.18		78.11		21.89
	4 = student	10,350	28.23		53.04		46.96
	5 = housewife	8136	22.19		2.94		97.06
	6 = not employed	3105	8.47		50.60		49.40
	7 = not yet in school	2578	7.03		50.81		49.19
**Covariates household level**							
caste	1 = no/ other/ general caste	7168	19.62		50.70		49.30
	2 = Scheduled caste/ tribe	15,969	43.70		51.41		48.59
	3 = Other backward class	13,404	36.68		51.99		48.01
	*missing*	*124*					
religion	1 = hindu	24,556	66.97		52.17		47.83
	2 = muslim	6586	17.96		50.47		49.53
	3 = christian/ other	5523	15.06		49.67		50.33
Below povery line status	1 = yes	23,956	65.34		51.68		48.32
	2 = no	12,709	34.66		51.11		48.89
		Mean	SD	Mean	SD	Mean	SD
household size	1 to 19 (continuous)	5.573	2.078	5.564	2.076	5.584	2.081
wealth index	1 = 1st quintile = poorest	6542	17.84		50.78		49.22
	2 = 2nd quintile	7370	20.10		51.79		48.21
	3 = 3rd quintile	7513	20.49		50.99		49.01
	4 = 4th quintile	7398	20.18		51.87		48.13
	5 = 5th quintile = least poor	7842	21.39		51.89		48.11
state	1 = Bihar	4855	13.24		53.16		46.84
	2 = Uttarakhand	4593	12.53		52.34		47.66
	3 = Uttar Pradesh	5322	14.52		52.82		47.18
	4 = West Bengal	3573	9.74		51.55		48.45
	5 = Gujarat	5167	14.09		52.06		47.94
	6 = Kerala	4287	11.69		47.42		52.58
	7 = Mizoram	4468	12.19		49.75		50.25
	8 = Tripura	4400	12.00		52.11		47.89

Missing values were automatically dropped from the analysis. Absolute numbers are reported for the sample size, outcome and key exposure variables

Abbreviations: hoh = head of household, SD = standard deviation, OOPE = out-of-pocket expenditure

Table 2 includes an overview of the sex-specific distribution of hospitalisation services sought after by persons who participated in this study. For our analysis, we summarised deliveries (*n* = 93) and Caesarean sections (*n* = 10) as maternal health (MH) services (*n* = 103). Overall, 35.3% of services for men and 28.7% for women were classified as “Others” because they could not be categorised based on the available data.

**Table 2:** Sex-specific distribution of hospitalisation services

		Maternal health Services	C-section	Delivery	Other services	Accident/ injuries	Arthritis	Asthma	Cancer	Cardiac treatment	Cataract	Cholera	Dengue	Diabetes	Diarrhea	Fever	Gastric treatment	Gynaecological treatment	Hypertension	Kidney	Malaria	Paralysis	Pneumonia	Typhoid	Others*	Total
Men	*n*		-	-		75	15	23	15	46	16	11	3	10	32	30	10	-	15	20	29	13	5	27	215	610
	%		-	-		12.3	2.46	3.77	2.46	7.54	2.62	1.8	0.49	1.64	5.25	4.92	1.64	-	2.46	3.28	4.75	2.13	0.82	4.43	35.25	100
Women	n		10	93		32	17	17	52	22	17	7	11	14	27	31	10	50	19	12	20	5	7	34	204	711
	%		1.41	13.08		4.5	2.39	2.39	7.31	3.09	2.39	0.98	1.55	1.97	3.8	4.36	1.41	7.03	2.67	1.69	2.81	0.7	0.98	4.78	28.69	100
Total	n		10	93		107	32	40	67	68	33	18	14	24	59	61	20	50	34	32	49	18	12	61	419	1321

* The “Others” category includes services which either have a very low sample size or were categorised as “others” already during the data collection process

### Analytical approach

To estimate the association between RSBY enrolment, health service utilisation and OOPE, we differentiated between hospitalisations for all services, including MH services, and hospitalisations for non-MH services. This means that we ran each analysis twice: first, on the sample that included hospitalisations for all services, and second, for the sample including only non-MH services.

We used descriptive statistics to illustrate the distribution of the outcome variables and to identify how many individuals (both men and women) were hospitalised. We conducted bivariate analyses to explore the distribution of the covariates among hospitalised and non-hospitalised individuals. For the OOPE analyses, we only included individuals who had a hospitalisation in the last year (i.e., 1321 individuals). We did not consider individuals’ second and third hospitalisation episodes in the analysis due to extensive missing data regarding OOPE. This resulted in 22 hospitalisation episodes being excluded from each regression model: 20 s hospitalisation episodes from 20 individuals and two episodes from the individual who was hospitalised three times. Additional sensitivity analysis (with 1321 individuals and 1343 hospitalisation episodes) showed that the estimates for the key exposure variables remained unchanged, supporting this approach (see supplementary files, Table S2). We calculated the mean and standard deviation (SD), and applied t-tests and analyses of variance (ANOVA) to examine differences among the variable categories.

We estimated two regression models: first, a multivariate logistic regression for *hospitalisation*, where we measured the likelihood of an individual, situated within a given household, being hospitalised during the past year. The model was expressed as follows:

$$\eqalign{& {\rm{Prob }}\left( {{\rm{y = 1}}} \right){\rm{ = }}{\beta _{\rm{0}}}{\rm{ + }}{\beta _{{\rm{sexind}}}}{\rm{ + }}{\beta _{{\rm{rsby}}}} \cr & \,\,\,{\rm{ + }}{\beta _{{\rm{sex*rsby}}}}{\rm{ + }}{\beta _{{\rm{covind}}}}{\rm{ + }}{\beta _{{\rm{covhh}}}}{\rm{ + }}\varepsilon \cr} $$

*y* was the binary outcome variable taking the value ‘1’ for the hospitalisation of an individual and ‘0’ for no hospitalisation in the past year. *sexind* (sex of the individual) and *rsby* (enrolment in RSBY) were the main exposure variables, as we assumed that hospitalisation depended on the individual’s sex and enrolment status in RSBY. We also included the interaction term sex*rsby to understand if, and to what extent, the association between RSBY and hospitalisation was different for men and women. We included a wide set of socio-economic characteristics at the individual (*covind*) and household (*covhh*) levels to control for possible confounders.

Second, as the outcome OOPE was highly skewed, we fitted a generalised linear model (GLM) gamma regression with a log link function. GLMs with gamma regression modelling techniques provide more accurate estimates of population means of healthcare expenditure than other models, and they accommodate skewness [52, 53]. The model was expressed as follows:

$$\eqalign{& ln\left[ {E\left( {Y|X} \right)} \right]{\rm{ }} = {\beta _0} + {\beta _{sexind}} + {\beta _{rsby}} \cr & \,\,\, + {\beta _{sex*rsby}} + {\beta _{covind}} + {\beta _{covhh}} + \varepsilon \cr} $$

ln[E(Y|X)] was the log link function for the outcome OOPE. We included the same exposure variables, covariates and interaction term as in the first model. For all analyses, standard errors were clustered at the household level, as individuals living in the same household share similar characteristics. We used the Wald test to assess variable inclusion and the Likelihood Ratio Test (LRT) to test for interactions and final model specification. The Wald test (*p* < 0.001) provided confirmation that the inclusion of the selected variables enhanced the statistical fit of the two regression models. The LRT ran each of our regression models twice: first without the interaction sex*rsby and second with the interaction. The LRT for the GLM regression confirmed significance (*p* = 0.0994), indicating that including the interaction term improved the final fit of the model. Despite the lack of significance in the LRT for the logistic regression, we included the interaction term in the final model for consistency in data analysis and reporting between the two models. All analyses were conducted using Stata 16.1 [54]. Initially, we also intended to analyse women’s health service utilisation and financial protection across the age groups used in this paper. We therefore stratified the bivariate analyses by these age groups, namely 0–14, 15–29, 30–49 and 50+. Results are consistent with those of the main analyses and are included as supplementary files (Tables S4 and S5). We also checked whether the outcome variables display different patterns for men and women who had accessed services in public or private facilities. Again, the results (see supplementary files, Table S6) are consistent with the main analyses. Additionally, we assessed the possibility of enrolment-related selection bias among women. First, we estimated a logistic regression model for hospitalisation among women and calculated the average marginal effects of RSBY enrolment. Second, to distinguish differences in utilisation from differences in financial protection, we estimated OOPE conditional on hospitalisation. We found no significant association between RSBY enrolment and hospitalisation, nor significant differences in OOPE conditional on service use, suggesting that the main findings are unlikely to be driven by differential healthcare utilisation among insured women (see supplementary files, Table S7).

## Results

Given our analytical focus, we show the complete model results in the supplementary files, but report the results of the main exposure variables, *sex* and *enrolment in RSBY*, exclusively.

### Outcome 1: Hospitalisation

Out of the total sample of 36,665 individuals, 51.1% of men and 48.9% of women were enrolled in RSBY. Overall, 1321 (3.6%) individuals were hospitalised, out of which 46.2% were men and 53.8% were women. Out of the hospitalised individuals, 46.8% of men and 53.2% of women were enrolled in RSBY.

When not taking enrolment in RSBY into account, bivariate analyses (Table 3) suggested significant differences between the outcome *hospitalisation* and the exposure variable *sex*. In general, more women were hospitalised than men (women: 4%, men: 3.23%, *p* < 0.001). However, this difference was not observed for non-MH hospitalisations. Hospitalisation rates were consistently higher among RSBY enrollees, regardless of whether we considered the sample including all services (enrolled: 4.13%, non-enrolled: 3.08%, *p* < 0.001) or the sample excluding MH services (enrolled: 3.86%, non-enrolled: 2.81%, *p* < 0.001).

**Table 3:** Bivariate analyses for *hospitalisation* and *OOPE*

	Hospitalisation								OOPE					
*n* = 36,665	All services				Non-MH services				All services			Non-MH services		
											p-value t-test or ANOVA			p-value t-test or ANOVA
	Total	hosp	%	Chi 2	Total	hosp	%	Chi 2	mean	SD		mean	SD	
ind sex														
male	18,877	610	3.23	0.000	18,877	610	3.23	0.272	4236	5390	0.0065	4236	5390	0.0139
female	17,788	711	4.00		17,685	608	3.44		3453	4725		3494	4770	
ind enrolment														
no	18,847	581	3.08	0.000	18,794	528	2.81	0.000	3929	5127	0.4992	4006	5064	0.4323
yes	17,638	729	4.13		17,588	679	3.86		3733	5035		3765	5194	

This table only shows the results for sex and enrolment, the key exposure variables. Full model results are shown in the supplementary files (Table S8)

Abbreviations: MH = maternal health, ind = individual, hosp = hospitalisation, OOPE = out-of-pocket expenditure, SD = standard deviation, PCC = pearson correlation coefficient

Once we differentiated between men and women enrolled and not enrolled in RSBY (Table 4), we observed that more women were hospitalised than men, regardless of their enrolment status (enrolled women: 4.5%, enrolled men: 3.78%, *p* = 0.016; non-enrolled women: 3.48%, non-enrolled men: 2.71%, *p* = 0.002). Again, this difference was not observed for non-MH hospitalisations.

**Table 4:** Bivariate analysis for hospitalisations of persons enrolled/ not enrolled in RSBY

		All services				Non-MH services		
*n* = 36,665	Total	hosp	%	Chi2	Total	hosp	%	Chi2
enrolled in RSBY								
male	9019	341	3.78	0.016	9019	341	3.78	0.574
female	8619	388	4.50		8569	338	3.94	
not enrolled in RSBY								
male	9766	265	2.71	0.002	9766	265	2.71	0.408
female	9081	316	3.48		9028	263	2.91	

Abbreviations: MH = maternal health, hosp = hospitalisation

The results from the multivariate logistic regression (Table 5) revealed that there was no statistically significant difference regarding the outcome variable hospitalisation between men and women, even after accounting for enrolment in RSBY. The results were consistent whether one considered the overall hospitalisation sample for all services or the one excluding hospitalisations related to MH services.

**Table 5:** Multivariate logistic regression for *hospitalisation*

*n* = 36,665	All services						Non-MH services					
	AOR	SE	95% CI		*p*		AOR	SE	95% CI		*p*	
ind sex												
male	1						1					
female	0.96	0.10	0.78	1.18	0.692		0.85	0.09	0.69	1.04	0.121	
ind enrolment												
no	1						1					
yes	1.13	0.10	0.94	1.34	0.186		1.11	0.10	0.93	1.33	0.249	
sex # enrolment RSBY												
female # RSBY	0.93	0.11	0.75	1.17	0.550		0.96	0.11	0.76	1.21	0.705	

This table only shows the results for sex and enrolment, the key exposure variables. Full model results are shown in the supplementary files (Table S9)

Baselines for the interaction *sex* and *enrolment in RSBY*: male # no RSBY, male # RSBY, female # no RSBY

Abbreviations: MH = maternal health, AOR = adjusted odds ratio, SE = standard error, CI = confidence interval, p = p-value, ind = individual

*Significant at 10%, **Significant at 5%, ***Significant at 1%

### Outcome 2: OOPE

Out of 1321 hospitalised, 1236 (93.57%) individuals experienced OOPE, of which 46.44% were men and 53.56% were women. Among those who incurred OOPE, 663 (53.64%) were enrolled in RSBY, with 46.76% being men and 53.24% being women.

When not considering the RSBY enrolment status of individuals, bivariate analyses (Table 3) suggested significant differences in OOPE based on *sex*: women (mean: INR 3453, SD: INR 4725, *p* = 0.0065) experienced lower OOPE than men (mean: 4236, SD: INR 5390) for all services as well as for non-MH services (mean: INR 3494, SD: INR 4770, *p* = 0.0139).

Once we differentiated between the enrolment status of men and women, our analysis (Table 6) revealed a similar significant difference in OOPE for individuals in RSBY. Specifically, enrolled women (mean: INR 3194, SD: INR 4338, *p* = 0.0032) experienced lower OOPE than enrolled men (mean: INR 4346, SD: INR 5671) for all services and for non-MH services (mean: INR 3185, SD: INR 4305, *p* = 0.0042). This difference disappeared for men and women who were not enrolled in RSBY.

**Table 6:** Bivariate analysis for OOPE of persons enrolled/ not enrolled in RSBY

*n* = 1321	All services			Non-MH services		
			p-value t-test			p-value t-test
	mean	SD		mean	SD	
enrolled in RSBY						
male	4346	5671	0.0032	4346	5671	0.0042
female	3194	4338		3185	4305	
not enrolled in RSBY						
male	4111	5087	0.4356	4111	5087	0.6407
female	3773	5163		3897	5309	

Abbreviations: MH = maternal health, SD = standard deviation

The results of the GLM model are shown in Table 7. Regarding hospitalisations for all services and without taking RSBY enrolment into account, we found suggestive evidence that OOPE for women (coef = -0.23; *p* = 0.070) was lower than for men. Looking at the interaction term sex*rsby, the results suggest that RSBY did not result in overall reductions in OOPE for the population at large, but it did bring down OOPE for women specifically. While the downward pattern is apparent for hospitalisations in the analysis for all services and for non-MH services, a decrease in OOPE (coef = -0.25; *p* = 0.087) is suggested at the 10% level only for the sample of hospitalisations due to non-MH services.

**Table 7:** Generalised linear model (GLM) for OOPE

*n* = 1321	All services					Non-MH services				
	coef	95% CI		*p*		coef	95% CI		*p*	
ind sex										
male	0.00					0.00				
female	-0.23	-0.47	0.02	0.070	*	-0.21	-0.46	0.05	0.111	
ind enrolment										
no	0.00					0.00				
yes	-0.03	-0.25	0.19	0.784		-0.01	-0.23	0.21	0.927	
sex # enrolment RSBY										
female # RSBY	-0.19	-0.47	0.09	0.192		-0.25	-0.54	0.04	0.087	*

This table only shows the results for sex and enrolment, the key exposure variables. Full model results are shown in the supplementary files (Table S10)

Baselines for the interaction *sex* and *enrolment in RSBY*: male # no RSBY, male # RSBY, female # no RSBY

Abbreviations: MH = maternal health, coef = coefficient, CI = confidence interval, p = p-value, ind = individual

*Significant at 10%, **Significant at 5%, ***Significant at 1%

## Discussion

This study makes an important contribution to the literature by being one of the first to examine differences in utilisation and OOPE between men and women, further distinguishing between MH and non-MH services. The policy implications of our findings relate to the design of PFHI, as defined in this paper, namely, tax-funded, non-contributory financing arrangements with explicit benefit packages and strategic purchasing mechanisms.

Our key findings reveal, first, that irrespective of enrolment into RSBY, women had a higher use of healthcare services, but only when MH services were factored into the analysis. Second, the decision to utilise healthcare services was not associated with enrolment in RSBY, for both men and women. And third, RSBY was not associated with decreased OOPE for the population as a whole, but the suggestive finding indicates a possible association with greater financial protection among women who sought non-MH services. In the following, we present our findings as historical evidence that may inform the design and implementation of PM-JAY and discuss their implications in the wider Indian and global socio-economic contexts.

First, we note that – independent of enrolment in RSBY – hospitalisation rates were slightly higher for women than for men, but only if MH services, in our case c-sections and deliveries, were included in the analysis. This result reflects the efforts of the Indian Government to increase institutional deliveries in recent decades (increase from 41% in 2005-06 to 79% in 2015-16) [55]. Despite this progress, the use of facility-based care for deliveries in India is still dependent on the socio-economic position and the place of residence, including state and rural-urban locality [55–58].

Our results also revealed that once we excluded MH services from the analysis, the difference in the hospitalisation rate between men and women was lost. This means that, factoring out MH services, hospitalisations under RSBY were largely gender-neutral. A previous study also reported no gender difference in the likelihood of hospitalisations in the general population younger than 60 years, whereas males older than 60 years were more likely to be hospitalised [59]. Regarding PM-JAY, general medicine is the most utilised service for men and women, followed by general surgery and oncology for men and by MH services and oncology for women [60]. Despite MH services typically driving female enrolment rates and being the second most accessed service by women, women’s overall utilisation of PM-JAY remains lower than for men (48% for women and 52% for men) [61]. This is evident, for example, in the utilisation of general medicine from 2019 to 2021, where these services accounted for 35.5% among men and 26.2% among women across all services accessed [60]. These differences indicate that hospitalisations under PM-JAY still have significant progress to make in achieving gender equity or neutrality. Nonetheless, our result for RSBY with the difference in hospitalisation rates for women, depending on whether MH services are included or excluded in the analysis, holds important implications for the way the impact of health policies is evaluated. In order to measure whether a health policy has contributed to improving women’s health, it is essential to expand health utilisation rates beyond maternal health and consider the changing healthcare needs of both men and women throughout their lifecycles, especially for morbidities where men and women experience similar incidence rates.

Second, our findings revealed no statistically significant association between the utilisation of hospitalisation services and enrolment in RSBY among either men or women. Accordingly, the analysis did not provide evidence that RSBY was associated with improved access to secondary healthcare, as enrolment in RSBY did not appear to have led to more hospitalisations. In general, the service uptake of RSBY was low, with a hospitalisation rate ranging between 2% in urban areas and 9% in rural areas, but also higher among women due to the utilisation of MH services [27, 29, 35, 36, 62]. The novel finding of our study is that the hospitalisation decision under RSBY was largely gender-neutral for enrolled men and women, regardless of MH or non-MH services. Regarding enrolment in RSBY, previous studies have already demonstrated a limited or no gender bias and even higher enrolment for female-headed households despite the five-member cap per household, although results differ between states [27, 28, 47, 48]. Evidence from other Indian welfare schemes highlights that despite being enrolled or entitled to benefits, women have difficulties with access because of a lack of decision-making power, restricted mobility and limited access to resources [56, 63–65]. Maternal empowerment indicators, such as the ability to leave the house or economic independence, are positively correlated with women’s use of MH services under RSBY [38]. On the one hand, we assume that the gender-neutrality regarding enrolment and hospitalisation under RSBY might have been the result of pro-women built-in design features of RSBY, such as the mandatory enrolment of spouses and the coverage of maternal health services [66, 67]. On the other hand, the gender-neutral findings suggest that no statistically significant association was observed between RSBY enrolment and hospitalisation among men and women who sought healthcare services, despite these provisions. For a country such as India, where gender disparities in access to healthcare are well-documented [68–75], this is an important finding. Early evidence on the implementation of PM-JAY also indicates that having access to the scheme does not lead to increased hospitalisations for either men or women [76]. In order to improve access to health services for men and women, learnings from RSBY and other schemes need to be incorporated into both pillars of the Ayushman Bharat Initiative, namely PM-JAY on the one hand and the *Health and Wellness Centres* (HWCs), responsible for delivering primary healthcare, on the other hand. A gender-sensitive and responsive approach to health needs requires that HWCs and PM-JAY effectively address the health needs of men and women throughout their lifecycles, ensuring that appropriate treatments and health services ranging from primary to secondary and tertiary care are provided at the right time.

Third, more than 93% of men and women in our sample experienced some form of OOPE during hospitalisation. Irrespective of enrolment in RSBY, we observed that OOPE for women was generally lower than for men. We assume that households were inclined to allocate their limited financial resources toward men’s healthcare rather than women’s, or they sought more expensive and higher quality care for men than for women. This is in line with previous studies, which demonstrate that households were more likely to rely on OOPE and distressed financing to cover hospitalisation expenses for men rather than women, indicating that India’s gender disparities are also reflected in the financing of healthcare with large variations by state [72, 73, 77]. OOPE among women may also have been influenced by concerns about exhausting the INR 30,000 coverage limit imposed under RSBY. With the substantially higher coverage ceiling under PM-JAY, such considerations are likely to be less pronounced. When considering enrolment in RSBY in our analyses, we found no association between RSBY enrolment and hospitalisation-related OOPE among the general population. This finding is consistent with studies on RSBY already cited earlier, which underline the limited financial protection RSBY provided to its beneficiaries [29–34]. Further analyses suggested a possible association between RSBY and reduced OOPE among women who sought non-MH services. This might be explained by the fact that MH services in India are covered by several specific and partially overlapping programmes, which might have led women to use RSBY particularly for non-MH services. The suggestive association between RSBY and reduced OOPE among women who sought non-MH services highlights the broader healthcare needs of women beyond MH. Although this finding cannot be directly transferred to PM-JAY because the two schemes differ substantially in their design and implementation, it offers valuable lessons for strengthening PM-JAY. Regarding PM-JAY’s financial protection, existing evidence on its impact on OOPE is mixed. One study found that PM-JAY did not reduce OOPE of patients [78]. Another study reported a 13% reduction in OOPE among individuals who accessed services in private, but not in public facilities, with no differences between men and women [76]. In addition, MH services, alongside general medicine, are a main driver for women’s overall utilisation of PM-JAY [60]. However, evidence on the impact of the scheme on OOPE for women’s hospitalisations for MH and non-MH services does not yet exist.

Against this background, the suggestive findings from RSBY may offer useful considerations for a gender-responsive design of services offered through PM-JAY and the HWCs, particularly with regard to financial protection for women accessing non-MH services. In designing such an approach, first, it is crucial to prevent the misuse of PM-JAY for unnecessary or unintended procedures, as occurred in some cases under RSBY. For example, in RSBY, hysterectomies have been a common procedure utilised by women, leading to widespread reports of misuse [79]. For PM-JAY, this underscores the importance of comprehensive counselling on sexual and reproductive health for both women and men, as well as eliminating any form of profit incentive for healthcare facilities to perform unnecessary hysterectomies. Additionally, improved claim reviews and data management are required to assess the necessity of procedures, especially of younger women, as well as variations and distributions of performed procedures within and between states [80]. Second, when considering our findings alongside evidence from PM-JAY, it emphasises the need for the Indian Government to prioritise policy efforts aimed at promoting equity in the utilisation of services for both men and women, as disparities still exist [81]. PM-JAY still lags behind on crucial elements like equity in supply and utilisation, targeting vulnerable population groups, cost coverage and awareness [81, 82]. And third, our findings can also inform India’s efforts to manage and address the healthcare needs over the lifecycle. This could include, for example, the set-up of an effective referral system under the Ayushman Bharat Initiative which more closely links primary, secondary and tertiary care under the HWCs and PM-JAY. Such a referral system would also support the Indian Government’s efforts to fight the growing burden of NCDs by providing comprehensive primary healthcare services through the HWCs [83]. The international literature on the role of health insurance, including PFHI, in the treatment of non-MH care is limited, including for India. A study using the 2002/03 World Health Survey data found that having all household members enrolled in mandatory or voluntary health insurance improved the likelihood of receiving treatment for chronic care by 38% [84]. A study from Brazil showed that individuals with access to health insurance were more likely to use NCD treatments [85].

Taken together, our results suggest that PFHIs, as non-contributory government-funded programmes with explicit entitlement packages and a shift from input-based to output-based purchasing, may contribute to reducing OOPE for women who seek treatment for non-MH services. We recommend additional qualitative research for PM-JAY and other PFHI to understand what causes such effects. This is especially important in the context of discussions on expanding health insurance to the “missing middle”, an estimated number of 300 to 400 million Indians (or 30% of the population) who currently lack any form of either publicly-funded or contributory health insurance [86, 87]. In this context, identifying which reforms to specific PFHI design features yield the greatest benefits for a given population is critically important. In addition, women’s access to PFHI in India still very much depends on multiple intersecting determinants like age, education, caste, and wealth [81, 88]. Findings should feed into reform processes of PFHIs in India and countries worldwide to ensure that these schemes are gender-responsive, equitable and universal in their design and implementation, and ultimately reach all poor and vulnerable. We further recommend research on the gendered effects of such financing arrangements that are substantially or entirely funded through general budget revenues, incorporate a purchaser-provider split, and provide an explicit benefit package. Such research should examine how the design and implementation of these financing arrangements, including the basis for entitlement, the definition and composition of the benefit package, and the availability and quality of services provided, influence gender equity in health service utilisation and financial protection. In addition, we recommend similar gender analyses of utilisation and financial protection under PM-JAY, incorporating an intersectional approach that considers factors like age, education, wealth, and other relevant dimensions of inequality.

### Methodological considerations

First, we used secondary data for which districts with high RSBY enrolment rates were purposively selected. This selection might have affected the distribution of enrolment in a non-random way. Although the sample may not be fully representative of all districts or populations, the sampling mechanism and unmeasured district-level factors may have affected the internal validity of the observed associations within the sample. It also limits external generalizability, as the findings are not nationally representative. Second, the available sample for the analysis of hospitalisations was small, which prevents our findings from being generalised to the whole of India or to settings that are different from the study states. We further acknowledge that these findings may vary across fully non-contributory schemes like the PM-JAY, nominally contributory schemes like the RSBY, and other schemes with varying levels of beneficiary- and state contributions, as the manner and amount of contribution can significantly affect demand for enrolment and beneficiary utilisation behaviour, especially among low-income or otherwise vulnerable households [89]. Therefore, we recommend studies which explore gendered differences in utilisation and OOPE in other settings with different contributory and subsidisation mechanisms. Third, we cannot account for the need for hospitalisations, meaning we do not know who in our sample fell sick and who among the sick was hospitalised. Fourth, one could speculate that our analysis is biased as it does not account for the distribution of service use between men and women, hence we cannot attribute observed differences in financial protection to the scheme, but rather to the underlying service use between enrolled men and enrolled women. Our descriptive statistics, however, do not detect major differences in the distribution of service use between enrolled men and enrolled women, hence defeating this argument. Fifth, although hysterectomies have been prevalent in India for years and are a leading reason for women to use health insurance [79], they were not included as a separate hospitalisation category in the household survey used for this study. We recommend research that specifically analyses the use of hysterectomies under PM-JAY. In addition, the survey does not include antenatal or postnatal services as separate categories or a further specification of the categories on gynaecological treatments and on cancer. Furthermore, although we have tried to account for the sex-specific distribution of hospitalisation services, we recognise that due to the self-reported data, 35.3% of services for men and 28.7% of services for women cannot be classified since they were categorised as “Others”. This is a common limitation of self-reported population-based data. Sixth, the data were collected in 2014, and RSBY is no longer operational. Nonetheless, the analysis of these data still delivers important practical lessons that can feed into the design and implementation of PFHI in India and other LMICs. Seventh, although state-level differences in social and gender-related factors might have affected RSBY enrolment, our sample was too small for subgroup analyses with adequate power. We therefore did not conduct more disaggregated analyses. We also acknowledge that clustering standard errors at the household level does not fully reflect the multi-stage sampling design. Because cluster-level adjustments were unavailable, our variance estimations may underestimate standard errors by assuming independent observations rather than accounting for shared cluster traits. Furthermore, because survey weights were unavailable, our point estimates may be biased and fail to reflect the true population distribution. Consequently, the representativeness and generalizability of the findings to the wider population may be limited. To account for systematic differences across states, we included state fixed effects in the regression models. And eighth, because information on the third gender was unavailable, we used *sex* to distinguish biological differences between males and females, and *gender* to capture socially constructed roles associated with men and women.

## Conclusion

Our study is one of the first to analyse the effect of a PFHI on women’s use of hospitalisation services and related OOPE while differentiating between MH and non-MH services. Although some of our results are suggestive, they still have important implications: first, PFHI coverage, as defined in this paper, may contribute to reduced OOPE for hospital-based non-MH services in LMICs, particularly among women, although further evidence is needed to establish this relationship. Second, if the health outcomes of women are to be improved, it is imperative that studies on PFHIs and other health programmes consider utilisation rates that go beyond maternal health. The focus should be on the provision of adequate, quality services that cater to the specific needs of both men and women. And third, our results contribute to the evidence base on gender-responsive health systems and ultimately on achieving UHC. The establishment of a universal health scheme alone is not enough to guarantee universal and equitable access, especially in countries that are characterised by large gender inequalities [3, 4, 11, 15, 16]. Universal schemes such as PFHIs may unintentionally replicate or even worsen gender inequalities in healthcare access, unless the schemes explicitly account for tackling gender inequalities that exist at the societal level through a gender-responsive design and implementation [3, 11, 15, 16, 90, 91]. The results from this paper suggest that gender considerations need to be integrated into PFHIs in India if access to healthcare is to be improved for women and girls.

## Supplementary Information

Below is the link to the electronic supplementary material.

Supplementary Material 1

## Data Availability

The original data are available with the Deutsche Gesellschaft für Internationale Zusammenarbeit (GIZ) GmbH in New Delhi, India. GIZ carried out the original data collection on behalf of the Indian Government (Ministry of Labour and Employment). The data are not publicly available. The authors obtained written permission from GIZ to use the data for the purpose of this study and GIZ shared the data with the authors in fully anonymised format. Data may be shared on request to the corresponding author with permission of GIZ.

## Abbreviations

GLM: Generalised linear model
HWC: Health and wellness centre
ID: Infectious disease
INR: Indian Rupee
LMIC: Low and middle income country
LRT: Likelihood Ratio Test
MCH: Maternal and child health
MH: Maternal health
NCD: Non-communicable disease
OOPE: Out-of-pocket expenditure
PFHI: Publicly-funded health insurance
PM-JAY: Pradhan Mantri Jan Arogya Yojana
RSBY: Rashtriya Swasthya Bima Yojana
UHC: Universal Health Coverage
