# Supplementary material for: Beyond maternal health: a gendered analysis of the associations between publicly-funded health insurance, service utilisation and financial protection among Indian women
Source: BMC Health Serv Res. 2026 Oct 5;26:1610. doi: 10.1186/s12913-026-15704-7 (PMC13770331; doi:10.1186/s12913-026-15704-7)
Supplement: Supplementary file 1 [file 12913_2026_15704_MOESM1_ESM.pdf]

Table S1: Key characteristics of RSBY and PM-JAY

	**RSBY** (data from 2016)	**PM-JAY** (current data)
Implementation period	2008 to 2018	Since 2018
Geographical coverage	In 25 states and union territories (out of 36)	in all 36 states and union territories
Eligible population	Households living below the poverty line. Total target population: 70 million households	Households who fulfill certain deprivation criteria in the Socio-Economic Caste Census of 2011. In total: 120 million households (or 550 million individuals [1]. Some states have expanded the eligibility criteria to other population groups or to their entire population.
Number of beneficiaries	41.2 million households or 134 million individuals (equals issued RSBY cards) [2,3]	435,2 million individuals (equals the number of issued cards which permits individuals to access services under PM-JAY – the so-called "Ayushman Cards") [4]
Unit of enrollment	Families, up to a maximum of 5 members	Families, no restriction on the family size
Level of care	Secondary, tertiary	Secondary, tertiary
Insurance coverage	30,000 Indian rupees per family per year (approx. 450 USD in 2016), transportation allowance ranging between 100 INR (approx. 1.5 USD in 2016) and 1,000 INR (approx. 15 USD in 2016) per hospitalization [5]	500,000 INR per family per year (approx. 6,000 USD)
Registration fee	30 INR (annually) [3]	None
Number of hospitalizations	Approx. 10.6 million from 2008 to 2016 [3]	Approx. 116,9 million [6]
Empaneled hospitals	Approx. 10,500 [3]	Approx. 36,000 [6]

Source: own depiction, refer to references in the table.

RSBY and PM-JAY have shared the objective of enhancing access to health care services and of decreasing out-of-pocket expenses for poor and vulnerable population groups. PM-JAY, like RSBY, is financed through general taxation from the central and state government and uses digital technology for implementation. In terms of benefits for the eligible population, PM-JAY has addressed some of the shortcomings of RSBY, for example, by expanding service and insurance coverage significantly. In order to access benefits, individuals were required to enroll in RSBY on an annual basis and they were issued an insurance card, the so-called RSBY card. PM-JAY is an entitlement-based scheme, where individuals automatically qualify based on certain deprivation criteria. They still need to undergo a verification process to prove their identity before receiving an "Ayushman card", the insurance card for PM-JAY.

References:

1 Mahal A, Panwar V, Tiwari A, *et al.* The ‘missing middle’: How to provide 350 million Indians with health coverage? *National Council of Applied Economic Research*. 2024;Working Paper No 162.

2 Karan A, Yip W, Mahal A. Extending health insurance to the poor in India: An impact evaluation of Rashtriya Swasthya Bima Yojana on out of pocket spending for healthcare. *Social Science & Medicine*. 2017;181:83–92. doi: 10.1016/j.socscimed.2017.03.053

3 Birdsall K. Health insurance for India’s poor: Meeting the challenge with information technology. Bonn: Deutsche Gesellschaft für Internationale Zusammenarbeit (GIZ) GmbH 2016.

4 Press Information Bureau, Government of India. Update on Progress of AB-PMJAY and ABDM. http://www.pib.gov.in/PressReleaseDetail.aspx?PRID=2241085&reg=3&lang=1 (accessed 20 May 2026)

5 Palacios R. A new approach to providing health insurance to the poor in India: the early experience of Rashtriya Swathya Bima Yojna. *India’s health insurance scheme for the poor. Evidence from the early experience of the Rashtriya Swasthya Bima Yojana.* New Delhi: Centre for Policy Research 2011:1–37.

6 Press Information Bureau, Government of India. Update on AB-PMJAY. http://www.pib.gov.in/PressReleaseDetail.aspx?PRID=2243769&reg=3&lang=1 (accessed 20 May 2026)

Table S2: Generalized linear model (GLM) for OOPE including individuals who were hospitalised twice and thrice

n=1321	All services				Non-MH services			
	coef	95%CI		p-value	coef	95%CI		p-value
ind sex								
…male	0.00				0.00			
…female	-0.28	-0.55	-0.01	0.044	-0.24	-0.52	0.04	0.093
ind enrolment								
…no	0.00				0.00			
…yes	0.01	-0.26	0.28	0.934	0.03	-0.23	0.30	0.798
sex # enrolment RSBY								
…female # RSBY	-0.26	-0.60	0.08	0.139	-0.33	-0.68	-0.02	0.062
age								
…0-14	0.00				0.00			
…15-49	-0.11	-0.51	0.28	0.572	-0.12	-0.52	0.27	0.542
…50+	-0.20	-0.64	0.24	0.374	-0.19	-0.63	0.25	0.392
relationship to hoh								
… hoh	0.00				0.00			
… spouse	0.40	0.02	0.78	0.041	0.48	0.07	0.89	0.021
… child	0.24	-0.10	0.58	0.164	0.27	-0.08	0.61	0.130
… others	0.41	0.06	0.77	0.022	0.35	-0.01	0.71	0.054
caste								
…No/ other/ general caste	0.00				0.00			
…scheduled caste/ tribe	-0.25	-0.54	0.04	0.094	-0.22	-0.53	0.08	0.145
…other backward class	-0.29	-0.59	0.02	0.063	-0.31	-0.62	0.00	0.051
ind religion								
…hindu	0.00				0.00			
…muslim	0.05	-0.26	0.36	0.766	0.08	-0.23	0.39	0.625
…christian/ other	-0.10	-0.62	0.43	0.720	-0.06	-0.58	0.45	0.808
ind education								
…no education	0.00				0.00			
…up to 7 years	-0.09	-0.32	0.13	0.423	-0.06	-0.30	0.17	0.603
…8 years and above	0.22	-0.04	0.48	0.098	0.25	-0.02	0.52	0.065
ind occupation								
…farming	0.00				0.00			
…labourer/ daily wages	-0.03	-0.36	0.31	0.876	0.00	-0.34	0.34	0.993
…other occup	0.24	-0.29	0.77	0.373	0.27	-0.27	0.81	0.330
…student	-0.73	-1.17	-0.29	0.001	-0.74	-1.19	-0.29	0.001
…housewife	-0.19	-0.60	0.23	0.372	-0.20	-0.64	0.25	0.385
…not employed	-0.18	-0.59	0.24	0.409	-0.15	-0.57	0.28	0.499
…not yet in school	-0.80	-1.40	-0.20	0.009	-0.78	-1.38	-0.18	0.011
below poverty line								
…yes	0.00				0.00			
…no	-0.08	-0.26	0.09	0.347	-0.09	-0.27	0.09	0.338
household size	0.03	-0.02	0.08	0.212	0.04	-0.02	0.09	0.171
wealth index								
…lowest	0.00				0.00			
…second	-0.09	-0.40	0.21	0.547	-0.11	-0.43	0.20	0.486
…middle	0.08	-0.24	0.41	0.606	0.09	-0.24	0.41	0.597
…fourth	-0.22	-0.51	0.07	0.142	-0.26	-0.56	0.03	0.083
…highest	0.09	-0.23	0.42	0.575	0.09	-0.23	0.42	0.571
state								
…Bihar	0.00				0.00			
…Uttarakhand	-0.17	-0.59	0.24	0.420	-0.23	-0.66	0.19	0.285
…Uttar Pradesh	0.23	-0.21	0.67	0.308	0.18	-0.27	0.62	0.442
…West Bengal	-0.17	-0.54	0.21	0.380	-0.22	-0.60	0.16	0.250
…Gujarat	-0.25	-0.62	0.12	0.185	-0.20	-0.59	0.20	0.326
…Kerala	0.48	-0.02	0.99	0.061	0.43	-0.07	0.92	0.095
…Mizoram	0.95	0.24	1.65	0.008	0.87	0.17	1.57	0.015
…Tripura	-0.08	-0.42	0.25	0.618	-0.21	-0.55	0.13	0.223

Abbreviations: MH = maternal health, ind = individual, hoh = head of household, OOPE = out-of-pocket expenditure.

Table S3: Generalized linear model (GLM) for OOPE with unwinsorised data

n=1321	All services				Non-MH services			
	coef	95%CI		p-value	coef	95%CI		p-value
ind sex								
...male	0.00				0.00			
...female	-0.28	-0.56	-0.01	0.041	-0.25	-0.53	0.03	0.078
ind enrolment								
...no	0.00				0.00			
...yes	-0.02	-0.28	0.25	0.908	0.01	-0.25	0.27	0.956
sex # enrolment RSBY								
...female # RSBY	-0.21	-0.54	0.13	0.223	-0.27	-0.61	-0.66	0.052
age								
...0-14	0.00				0.00			
...15-49	-0.12	-0.51	0.28	0.563	-0.13	-0.52	0.27	0.535
...50+	-0.26	-0.70	0.18	0.247	-0.25	-0.69	0.19	0.260
relationship to hoh								
... hoh	0.00				0.00			
... spouse	0.27	-0.08	0.63	0.134	0.35	-0.04	0.73	0.080
... child	0.22	-0.12	0.56	0.198	0.25	-0.09	0.59	0.153
... others	0.38	0.03	0.72	0.035	0.32	-0.03	0.68	0.075
caste								
...No/ other/ general caste	0.00				0.00			
...scheduled caste/ tribe	-0.24	-0.53	0.06	0.113	-0.21	-0.51	0.09	0.168
...other backward class	-0.28	-0.59	0.02	0.067	-0.31	-0.62	0.01	0.054
ind religion								
...hindu	0.00				0.00			
...muslim	0.02	-0.29	0.33	0.899	0.05	-0.27	0.36	0.768
...christian/ other	-0.11	-0.63	0.41	0.685	-0.07	-0.59	0.44	0.780
ind education								
...no education	0.00				0.00			
...up to 7 years	-0.13	-0.35	0.09	0.258	-0.10	-0.33	0.14	0.413
...8 years and above	0.22	-0.03	0.48	0.090	0.26	-0.01	0.53	0.057
ind occupation								
...farming	0.00				0.00			
...labourer/ daily wages	-0.04	-0.37	0.29	0.795	-0.02	-0.36	0.31	0.903
...other occup	0.05	-0.42	0.53	0.832	0.08	-0.41	0.57	0.757
...student	-0.75	-1.19	-0.30	0.001	-0.76	-1.21	-0.30	0.001
...housewife	-0.15	-0.56	0.27	0.487	-0.14	-0.59	0.31	0.537
...not employed	-0.14	-0.56	0.28	0.508	-0.11	-0.54	0.31	0.608
...not yet in school	-0.83	-1.43	-0.23	0.007	-0.81	-1.41	-0.20	0.009
below poverty line								
...yes	0.00				0.00			
...no	-0.07	-0.24	0.10	0.421	-0.07	-0.25	0.11	0.418
household size	0.03	-0.02	0.08	0.210	0.04	-0.02	0.09	0.177
wealth index								
...lowest	0.00				0.00			
...second	-0.10	-0.40	0.20	0.527	-0.12	-0.42	0.19	0.455
...middle	0.09	-0.23	0.40	0.587	0.09	-0.23	0.41	0.584
...fourth	-0.19	-0.47	0.10	0.199	-0.23	-0.52	0.06	0.117
...highest	0.14	-0.18	0.45	0.397	0.14	-0.18	0.46	0.395
state								
...Bihar	0.00				0.00			
...Uttarakhand	-0.17	-0.58	0.24	0.422	-0.23	-0.65	0.19	0.287
...Uttar Pradesh	0.21	-0.23	0.65	0.350	0.15	-0.29	0.60	0.498
...West Bengal	-0.17	-0.54	0.21	0.380	-0.22	-0.60	0.15	0.245
...Gujarat	-0.29	-0.66	0.07	0.118	-0.25	-0.63	0.14	0.215
...Kerala	0.51	0.00	1.01	0.050	0.45	-0.05	0.95	0.080
...Mizoram	0.94	0.25	1.64	0.008	0.86	0.16	1.56	0.016
...Tripura	-0.16	-0.49	0.17	0.328	-0.29	-0.63	0.05	0.095

Abbreviations: MH = maternal health, ind = individual, hoh = head of household, OOPE = out-of-pocket expenditure.

Table S4: Bivariate analyses for hospitalisation stratified by age group

	Ages 0-14					Ages 15-29								Ages 30-49								Ages 50+							
	All services				Non-MH services	All services				Non-MH services				All services				Non-MH services				All services				Non-MH services			
	Total	hosp	%	Chi 2	n.a.	Total	hosp	%	Chi 2	Total	hosp	%	Chi 2	Total	hosp	%	Chi 2	Total	hosp	%	Chi 2	Total	hosp	%	Chi 2	Total	hosp	%	Chi 2
ind sex																													
…male	5310	115	2.17	0.002		5608	109	1.94	0.000	5608	109	1.94	0.001	4665	171	3.67	0.003	4665	171	3.67	0.082	3294	215	6.53	0.635	3294	215	6.53	0.600
…female	5003	68	1.36			5007	220	4.39		4932	145	2.94		4716	232	4.92		1689	205	4.37		3063	191	6.24		3062	190	6.21	
ind enrolment																													
…no	6094	103	1.69	0.510		5668	175	3.09	0.994	5628	135	2.40	0.968	4237	159	3.75	0.024	4224	146	3.46	0.016	2848	144	5.06	0.000	2848	144	5.06	0.000
…yes	4129	77	1.86			4895	151	3.08		4860	116	2.39		5124	241	4.70		5110	227	4.44		3490	260	7.45		3489	259	7.42	
age																													
relationship to hoh																													
… hoh	-	-				630	22	3.49	0.000	630	22	3.49	0.000	3821	160	4.19	0.003	3820	159	4.16	0.026	3158	185	5.86	0.180	3158	185	5.86	0.179
… spouse	-	-				1097	87	7.93		1067	57	5.34		3655	185	5.06		3632	162	4.46		1529	98	6.41		1528	97	6.35	
… child	8391	152	1.81	0.554		7078	135	1.91		7066	123	1.74		1145	30	2.62		1144	29	2.53		95	5	5.26		95	5	5.26	
… others	1921	31	1.61			1810	85	4.70		1777	52	2.93		760	28	3.68		758	26	3.43		1575	118	7.49		1575	118	7.49	
caste																													
…No/ other/ general caste	1751	37	2.11	0.108		2024	63	3.11	0.561	2011	50	2.49	0.663	1958	111	5.67	0.001	1953	106	5.43	0.001	1435	106	7.39	0.090	1435	106	7.39	0.080
…scheduled caste/ tribe	4595	88	1.92			4685	136	2.90		4666	117	2.51		4071	148	3.64		4061	138	3.40		2618	148	5.65		2617	147	5.62	
…other backward class	3929	56	1.43			3871	128	3.31		3828	85	2.22		3323	143	4.30		3311	131	3.96		2281	148	6.49		2281	148	6.49	
ind religion																													
…hindu	6849	106	1.55	0.032		7027	240	3.42	0.002	6963	176	2.53	0.037	6347	281	4.43	0.000	6325	259	4.09	0.001	4333	289	6.67	0.002	4332	288	6.65	0.002
…muslim	1958	40	2.04			2000	62	3.10		1992	54	2.71		1587	85	5.36		1584	82	5.18		1041	78	7.49		1041	78	7.49	
…christian/ other	1505	37	2.46			1588	27	1.70		1585	24	1.51		1447	37	2.56		1445	35	2.42		983	39	3.97		983	39	3.97	
ind education																													
…no education	3851	93	2.41	0.001		1285	48	3.74	0.352	1271	34	2.68	0.501	3488	161	4.62	0.466	3474	147	4.23	0.502	3530	218	6.18	0.007	3530	218	6.18	0.005
…up to 7 years	5750	79	1.37			2703	84	3.11		2676	57	2.13		2534	107	4.22		2532	105	4.15		1934	147	7.60		1934	147	7.60	
…8 years and above	711	11	1.55			6627	197	2.97		6592	162	2.47		3359	135	4.02		3348	124	3.70		893	41	4.59		890	40	4.48	
ind occupation																													
…farming	53	0	0.00	0.001		1096	32	2.92	0.000	1091	27	2.47	0.000	2165	85	3.93	0.180	2163	83	3.84	0.538	1480	72	4.86	0.000	1480	72	4.86	0.000
…labourer/ daily wages	31	1	3.23			1814	40	2.21		1813	39	2.15		2457	96	3.91		2455	94	3.83		1134	67	5.91		1134	67	5.91	
…other occup	31	0	0.00			766	12	1.57		765	11	1.44		1010	38	3.76		1009	37	3.67		459	25	5.45		459	25	5.45	
…student	7200	101	1.42			3202	71	2.22		3201	70	2.19		28	0	0.00		28	0	0.00		12	2	16.67		12	2	16.67	
…housewife	124	1	0.81			2673	156	5.84		2609	92	3.53		3447	169	4.90		3425	147	4.29		1890	111	5.87		1889	110	5.82	
…not employed	384	9	2.34			1064	18	1.69		1061	15	1.41		274	15	5.47		274	15	5.47		1382	129	9.33		1382	129	9.33	
…not yet in school	2579	71	2.75			-	-			-	-			-	-			-	-			-	-			-	-		
below poverty line																													
…yes	6691	114	1.70	0.459		6970	213	3.06	0.721	6919	162	2.34	0.526	6032	249	4.13	0.282	6016	233	3.87	0.332	4263	279	6.54	0.462	4263	279	6.54	0.421
…no	3621	69	1.91			3645	116	3.18		3621	92	2.54		3349	154	4.60		3338	143	4.28		2094	127	6.06		2093	126	6.02	
wealth index																													
…lowest	2053	41	2.00	0.023		1678	42	2.50	0.052	1672	36	2.15	0.284	1677	61	3.64	0.191	1674	58	3.46	0.325	1134	59	5.20	0.105	1134	59	5.20	0.109
…second	2194	32	1.46			2038	48	2.36		2027	37	1.83		1881	69	3.67		1877	65	3.46		1257	70	5.57		1257	70	5.57	
…middle	2146	24	1.12			2185	76	3.48		2166	57	2.63		1892	88	4.65		1886	82	4.35		1290	98	7.60		1290	98	7.60	
…fourth	1964	41	2.09			2241	82	3.66		2218	59	2.66		1875	92	4.91		1866	83	4.45		1318	89	6.75		1317	88	6.68	
…highest	1955	45	2.3			2473	81	3.28		2457	65	2.65		2056	93	4.52		2051	88	4.29		1358	90	6.63		1358	90	6.63	
state																													
…Bihar	2018	22	1.09	0.000		1143	21	1.84	0.000	1139	17	1.49	0.000	970	39	4.02	0.000	965	34	3.52	0.000	724	35	4.83	0.000	724	35	4.83	0.000
…Uttarakhand	1285	13	1.01			1503	36	2.40		1496	29	1.94		1142	33	2.89		1140	31	2.72		663	32	4.83		663	32	4.83	
…Uttar Pradesh	1815	20	1.10			1570	40	2.55		1570	40	2.55		1143	30	2.62		1143	30	2.62		794	19	2.39		794	19	2.39	
…West Bengal	824	14	1.70			1006	41	4.08		1003	38	3.79		1093	54	4.94		1092	53	4.85		650	55	8.46		650	55	8.46	
…Gujarat	1288	17	1.32			1599	78	4.88		1561	40	2.56		1411	74	5.24		1397	60	4.29		869	67	7.71		869	67	7.71	
…Kerala	758	16	2.11			1057	24	2.27		1052	19	1.81		1338	49	3.66		1337	48	3.59		1134	106	9.35		1133	105	9.27	
…Mizoram	1287	28	2.19			1285	14	1.09		1284	13	1.01		1156	26	2.25		1155	25	2.16		746	19	2.55		746	19	2.55	
…Tripura	1043	53	5.08			1452	75	5.17		1435	58	4.04		1128	98	8.69		1125	95	8.44		777	73	9.40		778	73	9.40	
		mean	SD	t-test			mean	SD	t-test		mean	SD	t-test		mean	SD	t-test		mean	SD	t-test		mean	SD	t-test		mean	SD	t-test
household size		5.58	2.02	0.001			5.60	2.18	0.642		5.39	2.19	0.046		5.10	2.15	0.053		5.05	2.12	0.014		4.60	1.93	0.000		4.60	1.93	0.000

Abbreviations: MH = maternal health, ind = individual, hoh = head of household, hosp = hospitalisation, OOPE = out-of-pocket expenditure, SD = standard deviation, n.a. = not applicable.

Table S5: Bivariate analyses for OOPE stratified by age group

	Ages 0-14				Ages 15-29						Ages 30-49						Ages 50+					
	All services			Non-MH services	All services			Non-MH services			All services			Non-MH services			All services			Non-MH services		
	mean	SD	p-value t-test or ANOVA	n.a.	mean	SD	p-value t-test or ANOVA	mean	SD	p-value t-test or ANOVA	mean	SD	p-value t-test or ANOVA	mean	SD	p-value t-test or ANOVA	mean	SD	p-value t-test or ANOVA	mean	SD	p-value t-test or ANOVA
ind sex																						
…male	3363	4320	0.4762		3750	4818	0.5722	3750	4818	0.5758	5367	6306	0.0136	5367	6306	0.0398	4060	5292	0.0717	4060	5292	0.0717
…female	2891	4196			3421	4813		3397	4868		3890	5227		4079	5388		3163	4105		3163	4105	
ind enrolment																						
…no	3607	4872	0.1273		3970	5321	0.0822	4083	5472	0.0646	4646	5832	0.7515	4539	5810	0.5850	3330	4095	0.3185	3330	4095	0.3185
…yes	2602	3318			3010	4096		2920	3926		4453	5746		4539	5989		3844	5182		3844	5182	
age	-																					
relationship to hoh																						
… hoh	-				2760	2351	0.2204	2760	2351	0.5013	4508	5572	0.0130	4496	5588	0.0591	3847	5029	0.0299	3847	5029	0.0299
… spouse	-				2924	3911		2996	4074		3845	5231		4174	5475		3226	4199		3226	4199	
… child	3084	4052	0.4713		3492	4968		3638	5125		6751	6277		6934	6327		9564	9161		9564	9161	
… others	3710	5292			4398	5690		4268	5619		6890	8335		6522	8416		3397	4493		3397	4493	
caste																						
…No/ other/ general caste	2465	2545	0.0241		4350	5069	0.1491	4068	4944	0.4232	4968	6135	0.4858	5040	6241	0.4921	4112	5246	0.5091	4112	5246	0.5091
…scheduled caste/ tribe	4050	5389			3741	5125		3762	5233		4624	5825		4869	5963		3477	4171		3477	4171	
…other backward class	2197	2615			2930	4294		3021	4262		4082	5386		4174	5427		3435	4989		3435	4989	
ind religion																						
…hindu	3033	4124	0.2809		3450	4593	0.4277	3414	4464	0.3080	4332	5865	0.1395	4470	5984	0.1139	3362	4488	0.0095	3362	4488	0.0095
…muslim	2688	2583			3341	4558		3352	4721		4364	5126		4458	5194		3525	4677		3525	4677	
…christian/ other	4163	5829			4753	7025		5096	7438		6420	6141		6762	6178		5943	6449		5943	6449	
ind education																						
…no education	3217	4248	0.7473		3032	4186	0.5021	2814	4112	0.5929	3859	5104	0.0017	4001	5256	0.0013	3373	4209	0.4599	3373	4209	0.4599
…up to 7 years	3286	4559			3205	4778		3425	5051		3597	4407		3653	4437		4027	5628		4027	5628	
…8 years and above	2239	1726			3789	4967		3745	4920		5966	6958		6239	7073		3846	4713		3846	4713	
ind occupation																						
…farming	-		0.6304		2706	2517	0.0606	2615	2581	0.0255	4293	5060	0.1852	4307	5105	0.3083	4927	5501	0.0708	4927	5501	0.0708
…labourer/ daily wages	902	0			4608	5547		4639	5623		4982	5890		5038	5902		2721	4352		2721	4352	
…other occup	-				7279	8319		8032	8455		6313	7266		6365	7373		2001	2117		2001	2117	
…student	2897	3635			2800	4073		2800	4073		-						2513	3376		2513	3376	
…housewife	-				3468	4838		3369	4735		3924	5467		4163	5657		3437	4484		3437	4484	
…not employed	4354	6880			3881	5269		4290	5690		5632	7428		5632	7428		3851	5034		3851	5034	
…not yet in school	3481	4725			-			-			-											
below poverty line																						
…yes	2664	3483	0.0375		3519	4759	0.9560	3501	4669	0.8481	4508	5665	0.9529	4641	5733	0.9102	3720	5109	0.6437	3720	5109	0.6437
…no	4031	5215			3551	4922		3625	5146		4544	5905		4714	6059		3470	3998		3470	3998	
wealth index																						
…lowest	3223	4532	0.5893		3262	3975	0.3528	3660	4176	0.2805	4334	5590	0.0353	4407	5710	0.0259	3074	3585	0.7727	3074	3585	0.7727
…second	3527	5170			2945	4193		2783	4218		3069	4190		3186	4298		3680	4836		3680	4836	
…middle	4289	4706			3428	3814		3207	3362		5375	6192		5624	6286		3363	5292		3363	5292	
…fourth	2610	3795			3129	4951		3033	5008		3800	5231		3782	5224		3924	5289		3924	5289	
…highest	2825	3445			4478	6031		4641	6181		5576	6602		5815	6720		4001	4418		4001	4418	
state																						
…Bihar	2677	3275	0.0026		3115	3878	0.5032	3230	3991	0.5869	5010	6167	0.0686	5347	6435	0.1213	3651	5260	0.0360	3651	5260	0.0360
…Uttarakhand	1696	1553			4311	5754		4629	6025		4053	4846		4271	4934		2670	3219		2670	3219	
…Uttar Pradesh	6042	6623			3910	5074		3910	5074		4867	6836		4867	6836		2232	2615		2232	2615	
…West Bengal	3665	3641			3352	4136		3299	4259		4633	5547		4713	5572		3408	3838		3408	3838	
…Gujarat	1967	2005			2833	4396		3239	5306		2977	4894		3273	5286		3214	4638		3214	4638	
…Kerala	3164	5249			2230	3690		2608	4171		5716	7563		5812	7637		4219	6059		4219	6059	
…Mizoram	5029	6354			5318	7634		5611	7863		7207	6339		7418	6385		7121	6717		7121	6717	
…Tripura	2012	1655			3842	4743		2955	3353		4302	5006		4177	4905		3423	3735		3423	3735	
	PCC		p-value		PCC		p-value	PCC		p-value	PCC		p-value	PCC		p-value	PCC		p-value	PCC		p-value
household size	0.10		0.1702		0.06		0.2778	0.06		0.3185	0.08		0.1332	0.10		0.0689	0.02		0.6762	0.02		0.6762

Abbreviations: MH = maternal health, ind = individual, hoh = head of household, hosp = hospitalisation, OOPE = out-of-pocket expenditure, SD = standard deviation, PCC = pearson correlation coefficient, n.a. = not applicable.

Table S6: Bivariate analyses for hospitalisations and OOPE of persons in public and in private hospitals

Public hospitals

	**Hospitalisation**								**OOPE**					
	**All services**				**Non-MH services**				**All services**			**Non-MH services**		
	Total	hosp	%	Chi2	Total	hosp	%	Chi2	mean	SD	p-value t-test	mean	SD	p-value t-test
enrolled in RSBY														
...male	8895	217	2.44	0.211	8895	217	2.44	0.811	3700	5231	0.0893	3700	5231	0.0986
...female	8463	232	2.74		8432	201	2.38		2913	3964		2907	3901	
not enrolled in RSBY														
...male	9628	127	1.32	0.092	9628	127	1.32	0.801	3772	4890	0.3156	3772	4890	0.2688
...female	8909	144	1.62		8886	121	1.36		3177	4650		3104	4388	

Private hospitals

	**Hospitalisation**								**OOPE**					
	**All services**				**Non-MH services**				**All services**			**Non-MH services**		
	Total	hosp	%	Chi2	Total	hosp	%	Chi2	mean	SD	p-value t-test	mean	SD	p-value t-test
enrolled in RSBY														
...male	8804	126	1.43	0.013	8804	126	1.43	0.180	5370	6190	0.0072	5370	6190	0.01
...female	8392	161	1.92		8372	141	1.68		3558	4759		3562	4769	
not enrolled in RSBY														
...male	9640	139	1.44	0.010	9640	139	1.44	0.396	4396	5253	0.8162	4396	5253	0.8231
...female	8937	172	1.92		8907	142	1.59		4252	5509		4547	5896	

Abbreviations: MH = maternal health, hosp = hospitalisation.

Table S7: Average Marginal Effect sensitivity analysis

**Outcome**	**Estimator**	**RSBY coef/ AME**	**95% CI**		**p**
Hospitalisation	Logit AME	0.005	-0.001	0.012	0.101
OOPE	OLS coef	-241.18	-1284.91	802.56	0.650

Table S8: Bivariate analyses for *hospitalisation* and *OOPE*

	Hospitalisation								OOPE					
n=36665	All services				Non-MH services				All services			Non-MH services		
	Total	hosp	%	Chi 2	Total	hosp	%	Chi 2	mean	SD	p-value t-test or ANOVA	mean	SD	p-value t-test or ANOVA
ind sex														
...male	18877	610	3.23	0.000	18877	610	3.23	0.272	4236	5390	0.0065	4236	5390	0.0139
...female	17788	711	4.00		17685	608	3.44		3453	4725		3494	4770	
ind enrolment														
...no	18847	581	3.08	0.000	18794	528	2.81	0.000	3929	5127	0.4992	4006	5064	0.4323
...yes	17638	729	4.13		17588	679	3.86		3733	5035		3765	5194	
age														
...0-14	10312	183	1.77	0.000	10312	183	1.77	0.000	3186	4268	0.4992	3186	4268	0.4992
...15-29	10615	329	3.1		10540	254	2.41		3531	4810		3547	4840	
...30-49	9381	403	4.3		9354	376	4.02		4522	5753		4670	5854	
...50+	6357	406	6.39		6356	405	6.37		3643	4792		3643	4792	
relationship to hoh														
... hoh	7609	367	4.82	0.000	7608	366	4.81	0.000	4077	5176	0.2969	4070	5182	0.302
... spouse	6281	370	5.89		6227	316	5.07		3466	4693		3669	4888	
... child	16709	322	1.93		16696	309	1.85		3678	4905		3748	4971	
... others	6066	262	4.32		6031	227	3.76		4126	5552		3989	5464	
caste														
...No/ other/ general caste	7168	317	4.42	0.000	7150	299	4.18	0.000	4249	5327	0.0236	4228	5370	0.019
...scheduled caste/ tribe	15969	520	3.26		15939	491	3.08		4011	5220		4077	5269	
...other backward class	13404	475	3.54		13349	419	3.14		3302	4652		3362	4682	
ind religion														
...hindu	24556	916	3.73	0.000	24469	829	3.39	0.000	3642	4951	0.0013	3673	4975	0.000
...muslim	6586	265	4.02		6575	254	3.86		3626	4562		3657	4619	
...christian/ other	5523	140	2.53		5518	135	2.45		5347	6310		5487	6389	
ind education														
...no education	12154	520	4.28	0.000	12126	492	4.06	0.000	3463	4504	0.0056	3491	4547	0.000
...up to 7 years	12921	417	3.23		12892	388	3.01		3600	4955		3677	5014	
...8 years and above	11590	384	3.31		11544	338	2.93		4533	5780		4637	5860	
ind occupation														
...farming	4794	189	3.94	0.000	4784	182	3.80	0.000	4263	4947	0.0346	4291	5011	0.000
...labourer/ daily wages	5435	204	3.75		5432	201	3.70		4132	5411		4157	5432	
...other occup	2266	75	3.31		2264	73	3.22		5117	6595		5200	6675	
...student	10353	174	1.68		10353	173	1.67		2854	3792		2854	3792	
...housewife	8133	436	5.36		8049	349	4.34		3639	5006		3733	5082	

…not employed	3105	172	5.54		3101	169	5.45		4030	5364		4056	5391	
…not yet in school	2579	71	2.75		2579	71	2.75		3481	4725		3481	4725	
below poverty line														
…yes	23956	855	3.57	0.633	23889	788	3.30	0.000	3750	5029	0.5350	3785	5041	0.438
…no	12709	466	3.67		12673	430	3.39		3937	5114		4015	5214	
wealth index														
…lowest	6542	203	3.10	0.000	6533	194	2.97	0.000	3516	4526	0.0581	3607	4600	0.009
…second	7370	219	2.97		7355	204	2.77		3303	4535		3332	4594	
…middle	7513	286	3.81		7488	261	3.49		4099	5261		4151	5318	
…fourth	7398	304	4.11		7365	271	3.68		3490	4992		3484	4997	
…highest	7842	309	3.94		7821	288	3.68		4430	5543		4517	5589	
state														
…Bihar	4855	117	2.41	0.000	4846	108	2.23	0.000	3793	5056	0.0001	3893	5177	0.0002
…Uttarakhand	4593	114	2.48		4584	105	2.29		3451	4533		3547	4602	
…Uttar Pradesh	5322	109	2.05		5322	109	2.05		4226	5593		4226	5593	
…West Bengal	3573	164	4.59		3569	160	4.48		3820	4522		3838	4563	
…Gujarat	5167	236	4.57		5115	184	3.60		2925	4495		3126	4832	
…Kerala	4287	195	4.55		4280	188	4.39		4301	6265		4408	6345	
…Mizoram	4468	87	1.95		4466	85	1.90		6171	6611		6275	6655	
…Tripura	4400	299	6.80		4380	279	6.37		3557	4258		3307	3886	
		mean	SD	t-test		mean	SD	t-test	PCC		p-value	PCC		p-value
household size		5.14	2.11	0.000		5.05	2.09	0.000	0.05		0.0659	0.06		0.0470

Abbreviations: MH = maternal health, ind = individual, hoh = head of household, hosp = hospitalisation, OOPE = out-of-pocket expenditure, SD = standard deviation, PCC = pearson correlation coefficient.

Table S9: Multivariate logistic regression for *hospitalisation*

n=36665	**All services**						**Non-MH services**					
	AOR	SE	95% CI		p		AOR	SE	95% CI		p	
ind sex												
…male	1						1					
…female	0.96	0.10	0.78	1.18	0.692		0.85	0.09	0.69	1.04	0.121	
ind enrolment												
…no	1						1					
…yes	1.13	0.10	0.94	1.34	0.186		1.11	0.10	0.93	1.33	0.249	
sex # enrolment RSBY												
…female # RSBY	0.93	0.11	0.75	1.17	0.550		0.96	0.11	0.76	1.21	0.705	
age												
…0-14	1						1					
…15-29	1.57	0.24	1.17	2.11	0.002	***	1.50	0.23	1.11	2.04	0.009	***

…30-49	1.47	0.25	1.05	2.07	0.025		1.88	0.35	1.31	2.69	0.001	
…50+	2.00	0.34	1.43	2.80	0.000	***	2.64	0.48	1.85	3.78	0.000	***
relationship to hoh												
… hoh	1						1					
… spouse	1.06	0.13	0.83	1.35	0.649		1.15	0.15	0.90	1.49	0.267	
… child	0.51	0.06	0.40	0.64	0.000	***	0.59	0.07	0.46	0.76	0.000	***
… others	0.83	0.10	0.66	1.04	0.105		0.87	0.10	0.69	1.10	0.248	*
caste												
…No/ other/ general caste	1						1					
…scheduled caste/ tribe	1.12	0.09	0.95	1.32	0.184		1.15	0.10	0.97	1.37	0.113	
…other backward class	1.04	0.09	0.88	1.22	0.686		1.05	0.09	0.88	1.25	0.610	
ind religion												
…hindu	1						1					
…muslim	1.12	0.09	0.95	1.31	0.175		1.16	0.10	0.99	1.37	0.070	*
…christian/ other	1.01	0.18	0.71	1.43	0.966		1.02	0.19	0.71	1.47	0.907	
ind education												
…no education	1						1					
…up to 7 years	0.97	0.08	0.83	1.14	0.737		0.95	0.08	0.81	1.12	0.579	
…8 years and above	0.89	0.08	0.74	1.07	0.226		0.85	0.08	0.70	1.03	0.088	**
ind occupation												
…farming	1						1					
…labourer/ daily wages	0.96	0.10	0.78	1.19	0.729		0.98	0.11	0.79	1.21	0.819	
…other occup	0.89	0.13	0.67	1.19	0.435		0.90	0.13	0.67	1.21	0.483	
…student	1.03	0.16	0.75	1.41	0.856		1.24	0.21	0.89	1.73	0.197	
…housewife	1.43	0.18	1.11	1.83	0.005	***	1.25	0.16	0.97	1.61	0.086	
…not employed	1.63	0.20	1.27	2.08	0.000	***	1.70	0.22	1.32	2.18	0.000	***
…not yet in school	1.93	0.43	1.25	2.99	0.003	***	2.34	0.54	1.49	3.68	0.000	***
below poverty line												
…yes	1						1					
…no	1.11	0.07	0.98	1.25	0.100		1.11	0.07	0.97	1.26	0.127	
household size	0.95	0.02	0.92	0.99	0.006	***	0.93	0.02	0.89	0.97	0.000	***
wealth index												
…lowest	1						1					
…second	0.96	0.10	0.79	1.17	0.677		0.95	0.10	0.77	1.16	0.592	
…middle	1.26	0.12	1.04	1.53	0.017	**	1.23	0.12	1.01	1.50	0.037	
…fourth	1.36	0.13	1.13	1.64	0.001	***	1.30	0.13	1.07	1.59	0.008	***
…highest	1.31	0.13	1.08	1.59	0.005	***	1.34	0.13	1.10	1.63	0.004	***
state												
…Bihar	1						1					
…Uttarakhand	0.97	0.13	0.74	1.27	0.800		0.97	0.14	0.73	1.28	0.807	
…Uttar Pradesh	0.85	0.11	0.66	1.11	0.232		0.95	0.13	0.73	1.24	0.708	

…West Bengal	1.54	0.21	1.18	2.02	0.002	***	1.59	0.23	1.20	2.10	0.001	***
…Gujarat	1.69	0.19	1.35	2.12	0.000	***	1.44	0.18	1.13	1.84	0.003	***
…Kerala	1.52	0.20	1.16	1.97	0.002	***	1.54	0.22	1.17	2.03	0.002	***
…Mizoram	0.65	0.16	0.41	1.04	0.074	*	0.66	0.16	0.41	1.08	0.098	
…Tripura	2.56	0.31	2.02	3.25	0.000	***	2.54	0.32	1.98	3.26	0.000	***

Baselines for the interaction *sex* and *enrolment in RSBY*: male # no RSBY, male # RSBY, female # no RSBY.
Abbreviations: MH = maternal health, AOR = adjusted odds ratio, SE = standard error, CI = confidence interval, p = p-value, ind = individual, hoh = head of household.
*Significant at 10%, **Significant at 5%, ***Significant at 1%.

Table S10: Generalised linear model (GLM) for OOPE

n=1321	**All services**					**Non-MH services**				
	coef	95% CI		p		coef	95% CI		p	
ind sex										
...male	0.00					0.00				
…female	-0.23	-0.47	0.02	0.070	*	-0.21	-0.46	0.05	0.111	
ind enrolment										
…no	0.00					0.00				
…yes	-0.03	-0.25	0.19	0.784		-0.01	-0.23	0.21	0.927	
sex # enrolment RSBY										
…female # RSBY	-0.19	-0.47	0.09	0.192		-0.25	-0.54	0.04	0.087	*
age										
…0-14	0.00					0.00				
…15-29	-0.11	-0.49	0.27	0.561		-0.13	-0.51	0.25	0.498	
…30-49	0.17	-0.26	0.59	0.440		0.23	-0.20	0.66	0.299	
…50+	0.05	-0.36	0.46	0.813		0.12	-0.30	0.54	0.583	
relationship to hoh										
… hoh	0.00					0.00				
… spouse	0.11	-0.15	0.37	0.423		0.17	-0.10	0.45	0.223	
… child	0.26	-0.01	0.53	0.064		0.31	0.04	0.59	0.027	
… others	0.28	0.00	0.56	0.050		0.24	-0.04	0.52	0.093	
caste										
…No/ other/ general caste	0.00					0.00				
…scheduled caste/ tribe	-0.17	-0.40	0.07	0.161		-0.13	-0.36	0.11	0.289	
…other backward class	-0.23	-0.45	0.00	0.048	**	-0.24	-0.47	-0.01	0.041	**
ind religion										
…hindu	0.00					0.00				
…muslim	-0.07	-0.29	0.15	0.543		-0.03	-0.25	0.20	0.812	
…christian/ other	-0.06	-0.50	0.39	0.804		-0.02	-0.46	0.43	0.943	
ind education										

…no education	0.00					0.00				
…up to 7 years	-0.06	-0.26	0.13	0.523		-0.03	-0.23	0.18	0.789	
…8 years and above	0.21	-0.01	0.42	0.066	*	0.26	0.03	0.48	0.024	**
ind occupation										
…farming	0.00					0.00				
…labourer/ daily wages	-0.06	-0.34	0.21	0.647		-0.03	-0.31	0.25	0.851	
…other occup	-0.04	-0.41	0.33	0.823		-0.01	-0.39	0.37	0.950	
…student	-0.49	-0.89	-0.10	0.015	**	-0.45	-0.86	-0.04	0.030	**
…housewife	0.00	-0.30	0.31	0.979		0.02	-0.30	0.34	0.904	
…not employed	-0.09	-0.41	0.22	0.572		-0.04	-0.37	0.28	0.787	
…not yet in school	-0.51	-1.04	0.02	0.061	*	-0.43	-0.97	0.10	0.113	
below poverty line										
…yes	0.00					0.00				
…no	-0.05	-0.20	0.11	0.553		-0.05	-0.21	0.11	0.552	
household size	0.05	0.00	0.09	0.036	**	0.05	0.00	0.10	0.039	**
wealth index										
…lowest	0.00					0.00				
…second	-0.04	-0.30	0.23	0.784		-0.07	-0.33	0.20	0.626	
…middle	0.11	-0.13	0.36	0.375		0.10	-0.15	0.35	0.441	
…fourth	-0.10	-0.34	0.15	0.439		-0.14	-0.39	0.11	0.263	
…highest	0.13	-0.11	0.38	0.278		0.14	-0.11	0.38	0.281	
state										
…Bihar	0.00					0.00				
…Uttarakhand	-0.16	-0.52	0.20	0.390		-0.22	-0.58	0.14	0.238	
…Uttar Pradesh	0.14	-0.26	0.53	0.495		0.10	-0.30	0.49	0.626	
…West Bengal	-0.09	-0.43	0.24	0.592		-0.15	-0.48	0.19	0.396	
…Gujarat	-0.28	-0.61	0.05	0.092	*	-0.26	-0.61	0.09	0.142	
…Kerala	0.05	-0.32	0.42	0.798		-0.01	-0.38	0.36	0.970	
…Mizoram	0.67	0.08	1.25	0.025	**	0.57	-0.02	1.15	0.059	**
…Tripura	-0.08	-0.38	0.21	0.580		-0.22	-0.52	0.08	0.150	

Baselines for the interaction *sex* and *enrolment in RSBY*: male # no RSBY, male # RSBY, female # no RSBY.

Abbreviations: MH = maternal health, coef = coefficient, CI = confidence interval, p = p-value, ind = individual, hoh = head of household.

*Significant at 10%, **Significant at 5%, ***Significant at 1%.
